# Functional Characterization of *ycao* in *Escherichia coli* C91 Reveals Its Role in Siderophore Production, Iron-Limited Growth, and Antimicrobial Activity

**DOI:** 10.3390/antibiotics15010043

**Published:** 2026-01-01

**Authors:** Khadijah M. Dashti, H. Ebrahim, Leila Vali, Ali A. Dashti

**Affiliations:** 1 Department of Medical Laboratory Sciences, Faculty of Allied Health Sciences, Kuwait University, Kuwait City, Kuwait; ali.dashti2@ku.edu.kw; 2 Department of Biotechnology, School of Arts and Sciences, American International University, Jahra, Kuwait; h.ebrahim@aiu.edu.kw; 3 Department of Infection Biology & Microbiomes, Institute of Infection, Veterinary and Ecological Sciences, University of Liverpool, Liverpool, CH64 7TE, UK; 4 School of Education and Applied Science, Francis Close Hall Campus, University of Gloucestershire, Cheltenham GL50 2RH, UK; lvali@glos.ac.uk

**Keywords:** antibiotics, antimicrobials, multidrug-resistance, natural products, secondary metabolites

## Abstract

Background: The emergence of antibiotic-resistant bacteria is one of the top health concerns. *Escherichia coli* is a Gram-negative bacterium that commonly causes severe infections. However, this research exposed its antibiotic-producing potential. Methods: Rifampicin-resistant mutants of *E. coli* C91 were generated to activate cryptic BGCs. Mutants (C91-R1, R2 and R3) were tested for antimicrobial production using agar-well diffusion assays. Metabolite profiling was performed by LC-MS/MS. Siderophore production was tested by construction of a Δ*ycao* deletion mutant. Growth of this mutant was assessed under iron-limited conditions versus iron-rich conditions using dipyridyl. qRT-PCR was used to analyze gene expression *entB*, *mcmA* and *mchF*. Genome mining was performed using antiSMASH and BAGEL4. Results: Compared to the wild type, Mutant C91-R1(S531L) displayed clear antibacterial activity against *Staphylococcus aureus*. LC-MS/MS revealed unique metabolites, including a novel peak at *m*/*z* 410.5, specific to the mutant C91-R1. A reduction in siderophore production of 61% was demonstrated in the Δ*ycao* mutant, and downregulation of *entB*, *mcmA* and *mchF.* Conclusions: Genome mining predicted non-ribosomal peptide, thiopeptide and polyketide BGCs. *E. coli* C91 offers antibiotic-producing potential that can be activated through ribosome-engineering-type approaches. Moreover, *E. coli* C91-R1 has unique metabolites and is considered as a promising candidate for novel antibiotic discovery.

## 1. Introduction

The spread of antibiotic-resistant bacteria throughout healthcare facilities poses a significant challenge to patient safety. The ESKAPE pathogens represent a group of clinically significant Gram-positive and Gram-negative bacteria which demonstrate a rise in antibiotic resistance [1]. The ESKAPE pathogens consist of *Enterococcus faecium*, *Staphylococcus aureus*, *Klebsiella pneumoniae*, *Acinetobacter baumannii*, *Pseudomonas aeruginosa* and *Enterobacter* species. Healthcare facilities face a major threat from the spread of antibiotic-resistant bacteria, primarily due to the ESKAPE pathogens [2].

The Centers for Disease Control and Prevention (CDC) reported that methicillin-resistant *Staphylococcus aureus* (MRSA) was isolated from 30% of all bacterial samples that existed in multiple healthcare environments, including hospitals [3]. Moreover, this report also indicated that vancomycin-resistant enterococci (VRE) were a primary bacterial cause of bloodstream infections in different hospital settings. The World Health Organization (WHO) reported that *K. pneumoniae* and *A. baumannii* from Eastern Europe and Asian countries demonstrated more than 40–60% resistance to extended-spectrum β-lactams and carbapenems [4]. In addition, antibiotic resistance of *P. aeruginosa* and *Enterobacter* species continues to rise as they develop resistance to fluoroquinolones and colistin, with the latter serving as the last resort antibiotic [5]. The growing spread of multidrug-resistant (MDR) bacteria is pressuring researchers to explore new biosynthetic pathways for antimicrobial production [6].

Some bacteria have the capability to produce a wide range of natural products (NPs), including bacteriocins, [7]. Interestingly, some of these NPs or secondary metabolites (SM) exhibit antimicrobial properties [8]. Biosynthetic gene clusters (BGCs), which are a group of genes that together code to produce proteins involved in antimicrobial synthesis, control the biosynthesis of NPs [9]. Biosynthetic genes enable the co-expression of biosynthetic enzymes, regulators and transporters involved in NP production and secretion [9]. Researchers are now mining bacterial genomes for biosynthetic genes involved in antibiotic biosynthesis to accelerate antibiotic discovery [8]. Thousands of putative BGCs that can code for the production of novel antimicrobial compounds can be generated by genome mining; however, many BGCs will be nearly identical, and many will produce known compounds [10]. Several platforms for the automated detection of BGCs in a genome have been developed, with “antibiotics and secondary metabolites analysis shell” (antiSMASH) and “PRediction Informatics for Secondary Metabolomes” (PRISM) being two of the most popular and capable of recognizing novel NPs [11,12].

*Escherichia coli* is a Gram-negative bacterium that lives in the environment and plays a role both in nature and healthcare settings [13]. Commensal strains of this bacterium are commonly found in the gut; however, some have evolved pathogenic traits due to horizontal gene exchange and adaptability [14]. Most *E. coli* strains are harmless commensals; however, some of them, including uropathogenic *E. coli* (UPEC), possess multiple virulence factors that enable them to cause urinary tract infections [15]. Examples of virulence factors include adhesins such as P fimbriae, type 1 fimbriae and toxins such as hemolysins [16]. It also produces cytotoxins such as α-hemolysin and cytotoxic necrotizing factor 1 (CNF1), which modulate immune responses and damage host tissues [17]. Moreover, UPEC has the ability to overcome iron-restricted environments by facilitating iron acquisition systems, including siderophores such as enterobactin and yersiniabactin [17]. UPEC is a strain that commonly causes infections ranging from uncomplicated cystitis, recurrent UTIs to severe cases of urosepsis [18]. Previous genomic data has demonstrated that UPEC translocates from the host’s gastrointestinal reservoir to the periurethral area, causing UTIs [19]. Despite this, *E. coli* has been shown to exhibit competitive behavior in challenging settings, such as iron-limited environments [20]. *E. coli* employs a tactic when facing limited iron availability by producing siderophores, which are small iron-chelating molecules such as enterobactin, salmochelin and yersiniabactin [21]. These siderophores function by sequestering iron from both the environment and the host [22]. These mechanisms are regulated by the ferric uptake regulator (Fur), contributing significantly to virulence, biofilm formation and competition between bacteria. Studies have reported the connection of siderophore production by bacteria with secondary metabolite production [23]. When iron levels are low in the environment, it acts as an indicator to activate gene clusters involved in metabolite biosynthesis that would otherwise remain silent or inactive [23,24].

*E. coli* is one of the top bacteria with metabolic diversity; however, it is often overlooked as a producer of NPs [25]. *E. coli* can produce many NPs, including siderophores and polyketides; however, characterization of the antibacterial properties of these NPs remains to be explored. Previously, *E. coli* has been utilized, and its carbapenem synthesis pathway was metabolically engineered to allow access to the full range of natural carbapenems [26]. Researchers have also investigated compounds produced by *E. coli* that can alter the antibiotic production pattern of *Streptomyces* sp. [27]. However, less effort has been made towards discovering *E. coli* BGCs that carry genes with the potential to code for unique NPs with antimicrobial properties.

The main aim of this study was to determine the antimicrobial properties of siderophores, and other antimicrobials produced by *E. coli* C91 using both bioinformatics-based and culture-based approaches.

## 2. Results

### 2.1. Production of Antimicrobials by E. coli C91 and E. coli C91-R1

**Figure 1:**
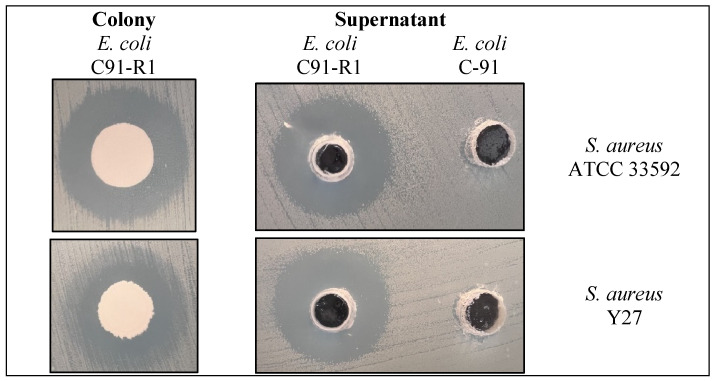
Inhibition formed by *E. coli* C91-R1 against two strains of *Staphylococcus aureus*.

Three spontaneous rifampicin-resistant *E. coli* C-91 colonies were isolated (C91-R1, C91-R2 and C91-R3). According to spot-on-lawn assays, only *E. coli* C91-R1 exhibited activity against indicator bacteria. This mutant was able to inhibit both *S. aureus* Y27 and *S. aureus* ATCC 33592 according to spot-on-lawn assay, suggesting antibiotic production (Figure 1).

The supernatant of mutant *E. coli* C91-R1 demonstrated the ability to suppress the growth of both *S. aureus* Y27 and *S. aureus* ATCC 33592 through agar-well diffusion assays (Table 1). Phenotypic stability of C91-R1 was evaluated by subculture of this bacterium for five consecutive passages under non-selective conditions. The phenotype was stable as demonstrated by consistent retained antibacterial activity against MRSA. Negative controls showed no inhibition zones. Positive controls produced reproducible zones of inhibition ranging from 21 to 27 mm for ciprofloxacin (10 µg/mL), and 18–24 mm for linezolid (10 µg/mL).

The *rpoB* gene sequencing of these mutants revealed mutations that occurred in the rifampicin resistance-determining region (RRDR) of the RNA polymerase β-subunit. The mutations S531L (*E. coli* C91-R1), H526Y (*E. coli* C91-R2), and D516V (*E. coli* C91-R3) were detected (Table 1).

### 2.2. LC-MS of Supernatants

LC-MS analysis was conducted as a qualitative metabolomic screen to identify strain-specific ionic features. Since this study focused on detection rather that quantification, peak intensities were not subjected to statistical analysis. Like other studies that focus on metabolic discovery, they focus on chemical identity rather than abundance [29].

Rif resistance-conferring *rpoB* mutations generated unique metabolome patterns with *E. coli* C91 culture supernatants as analyzed by LC–MS. There was no evident peak of secondary metabolites in the wild-type strain, but the three *rpoB* mutants (C91-R1, R2 and R3) produced specific biosynthetic pathways as indicated by ion features.

**Figure 2:**
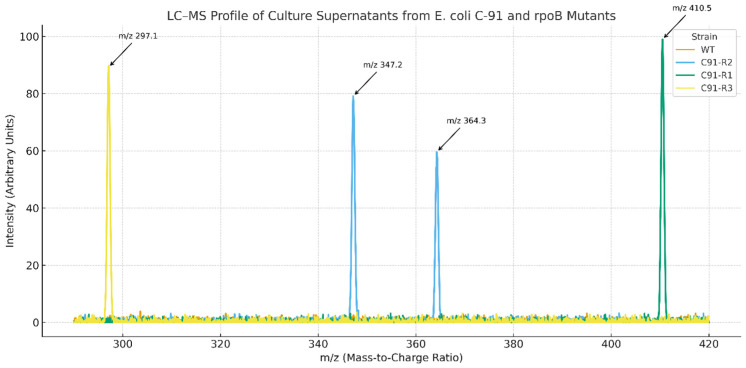
LC–MS profiling of culture supernatants from *E. coli* C-91 and *rpoB* mutants. Extracted ion chromatograms (EICs) showing strain-specific production of secondary metabolites. C91-R2 exhibited peaks at *m*/*z* 347.2 and 364.3; C91-R1 at *m*/*z* 410.5; and C91-R3 at *m*/*z* 297.1. The wild-type strain (WT) showed no significant signal under identical conditions. Data are representative of three independent experiments.

*E. coli* C91-2 showed significant peaks at *m*/*z* 347.2 and 364.3, which were not detected in the wild-type strain, reflecting to putative peptide-like metabolites (Table 2). A peak at *m*/*z* 410.5 presented only in a unique product from *E. coli* C91-R1 and not in other strains (Figure 2). In contrast, C91-R3 showed a unique *m*/*z* 297.1 ion feature that did not share the same profile with C9-R1 or C9-R2. These results suggest that individual *rpoB* mutations selectively activate cryptic biosynthetic pathways in *E. coli* C-91.

A tandem MS (MS/MS) fragmentation was carried out to further identify the metabolite at *m*/*z* 410.5 that we only observed in C91-R1. The subsequent fragmentation pattern featured a set of structurally diagnostic daughter ions at *m*/*z* 392.5, 374.4, 356.3, 328.2 and 300.1 indicative of the presence of polyene or non-ribosomal peptide backbone structure. In silico analysis using GNPS and METLIN did not identify known compounds to correspond to this spectrum, indicating the metabolite could be a new or an uncharacterized antimicrobial molecule (Table 2).

### 2.3. CAS Assay for Siderophore Production

To determine siderophore production, the chrome azurol S (CAS) agar diffusion assay was conducted for *E. coli* C91 wild type and Δ*ycao* mutant. The stability of CAS reagent was confirmed prior to each experiment. The positive control (*Pseudomonas aeruginosa* PAO1) produced orange halo, and uninoculated negative control retained the blue color. The halo produced by the wild-type strain had a mean diameter of 14.2 ± 0.4 mm, compared to the significantly smaller (5.6 ± 0.3 mm; mean ± s.d.; *n* = 3) halo formed by the Δ*ycao* mutant (61% less siderophore activity than wild type) (Table 3). The statistical analysis with unpaired two-tailed Student’s *t*-test revealed that this difference was statistically significant (*p* = 0.0002). Under the tested conditions, it was shown that the reduction in halo diameters (61%) in the Δ*ycao* mutant contributed significantly to siderophore production, which is consistent with a role in iron acquisition. These data indicate a function of *ycao* in siderophore biosynthesis or iron acquisition in *E. coli* C91.

### 2.4. Growth Under Iron-Limiting Conditions

**Figure 3:**
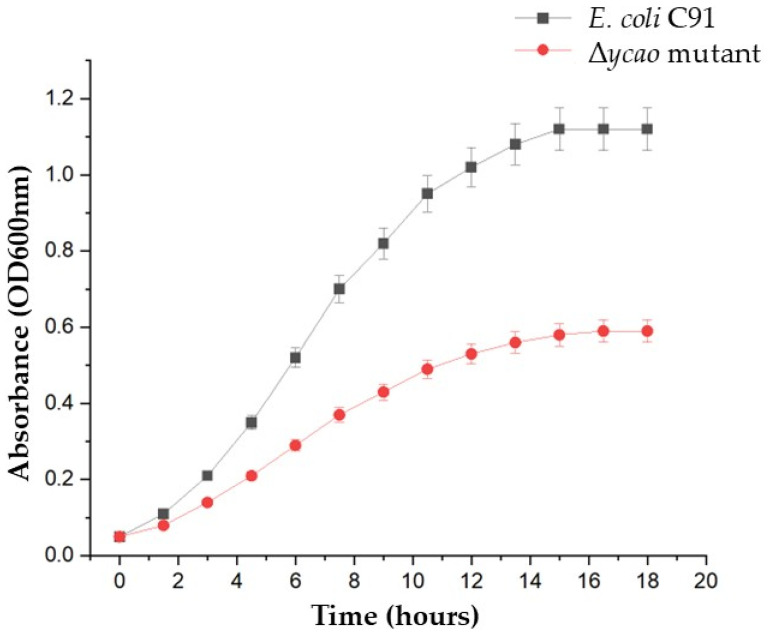
Growth of *E. coli* C91 and Δ*ycao* mutant under iron-limiting conditions.

The role of *ycao* in iron uptake was investigated by comparing *E. coli* C91 wild type and Δ*ycao* mutant strain grown in M9 minimal medium with or without the iron chelator 2,2–dipyridyl. Both strains displayed comparable growth kinetics and final optical density ((OD_600nm_): 1.12 ± 0.04 in wild type versus 1.09 ± 0.03 in Δ*ycao*; *p*-value= 0.48) when cultured under iron-replete conditions (Table 4). The Δ*ycao* mutant exhibited marked impairment of growth compared with the wild type, in response to iron depletion (100 µM dipyridyl) ([AUC; 5.54 ± 0.35 vs. 8.89 ± 0.15]; *p*-value = 0.0001, unpaired two-tailed *t*-test, *n* = 3) (Figure 3).

Both *E. coli* C91 wild type and Δ*ycao* mutant reached a similar final AUC under iron-replete conditions. Although the AUC values showed minor difference between the wild type and the Δ*ycao,* this difference was not statistically significant (*p* > 0.05, unpaired two-tailed *t*-test). Therefore, the deletion of *ycao* does not impair growth when iron is readily available in the growth medium. Conversely, the Δ*ycao* mutant strain showed reduced AUC and final OD under iron-limited conditions (*p* < 0.001), which indicates that the loss of *ycao* affects the growth *of E. coli* when iron is scarce, which is in line with its lower siderophore production capability.

### 2.5. Generation and Molecular Confirmation of the Δycao Mutant

To examine the functional relevance of the *ycao* gene in *E. coli* C91, an in-frame deletion mutant (Δ*ycao*) was obtained by λ-Red-mediated recombination. Sanger sequencing on the amplified products was used to ensure the molecular integrity of the deletion. Sequence comparison showed that the whole *ycao* coding region was successfully deleted, and the upstream and downstream flanking regions were seamlessly fused without exhibiting any polar effects or vector sequences. The sequences of the primers used, and complete nucleotide sequence data from wild type and Δ*ycao* loci, are available in Supplementary Table S2.

### 2.6. Transcription Analysis

To investigate if reduced production of siderophores in the Δ*ycao* mutant correlated with transcription level, a subset of biosynthetic and effector genes (*entB, mcmA* and *mchF*) were examined using quantitative real-time PCR (qRT-PCR). Gene expression was normalized to the housekeeping gene *rpoD* and is presented as relative compared with the wild-type *E. coli* C91 strain.

**Figure 4:**
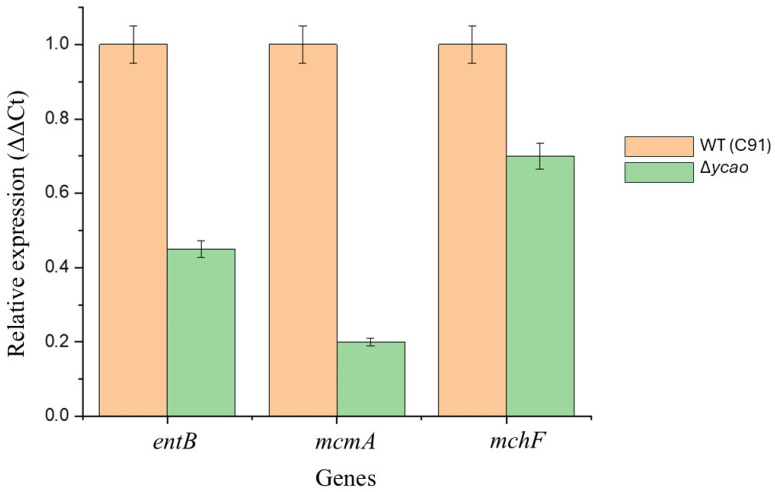
Transcriptional analysis by qRT-PCR. Figure shows relative expression levels of siderophore- and antibiotic-associated genes in the Δ*ycao* mutant compared to the wild-type *E. coli* C91 strain. Relative expression normalized to the housekeeping gene *rpoD*.

For the siderophore biosynthetic gene *entB*, a marked reduction was observed in expression in the Δ*ycao* mutant with a 2.22-fold decrease in transcript levels relative to WT when grown under iron-limited conditions. Of the known genes involved in microcin maturation and transportation, *mcmA* and *mchF* also demonstrated a similar trend of downregulation by 5-fold and 1.4-fold in the mutant, respectively (Figure 4). Raw qRT-PCR results corresponding to the bar graph are presented in the Supplementary Data Table S3. These results are consistent with the CAS assay defective phenotype. While this data shows simultaneous down regulation of siderophore (*entB*) and microcin-associated genes (*mcmA*, *mchF*), the experimental procedure does not directly demonstrate any functional changes in microcin production. Thus, the *ycao* deletion and microcin-associated pathways remain to represent a correlative relationship.

### 2.7. Prediction of Genes Coding for Possible Antibacterial Compounds in the Genome of E. coli C91

The antiSMASH, BAGEL4 and PRISM were used to predict secondary metabolite gene clusters in the genome of *E. coli* C91. The antiSMASH predicted two BGCs; one non-ribosomal peptide (NRP) BGC and one thiopeptide BGC. PRISM predicted a BGC with genes that could code for proteins involved in the biosynthesis of a polyketide (Table 5). BAGEL did not predict any BGCs in the WGS of *E. coli* C-91.

**Figure 5:**
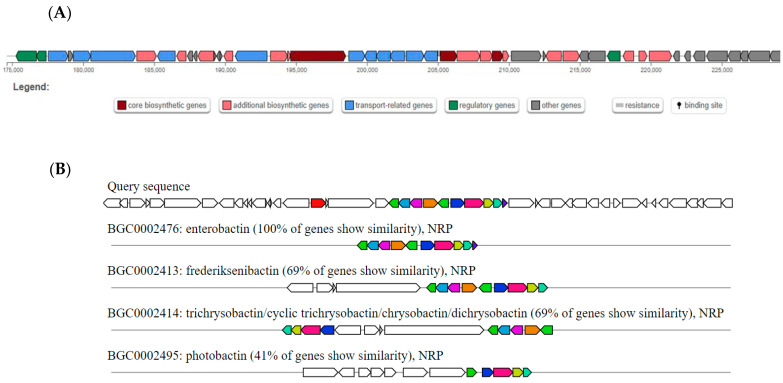
Non-ribosomal peptide BGC in the whole genome sequence of *Escherichia coli* C-91 as predicted by antiSMASH. (**A**) Diagrammatical representation of NRP BGC in the genome of *E. coli* C-91. (**B**) Comparison of known BGCs with the non-ribosomal peptide BGC in the whole genome sequence of *Escherichia coli* C-91 as predicted by antiSMASH. The figure represents a set of BGCs with genes that encode for proteins with a predicted function. These genes are also predicted in the query BGC. The query sequence is the putative NRP BGC in the WGS of *E. coli* C91. The percentage represents the percentage of genes in the BGCs that have a significant BLAST hit (version 2.12.0) to genes within the current region. All figures were adapted from antiSMASH version 7.1.0.

The antiSMASH predicted one non-ribosomal peptide (NRP) BGC to carry genes that encode proteins involved in the biosynthesis of enterobactin, a known metabolic product of *E. coli* (Figure 5A). In the WGS of *E. coli* C91, all of the genes of enterobactin BGC were identical to those of the NRP BGC (Figure 5B).

**Figure 6:**
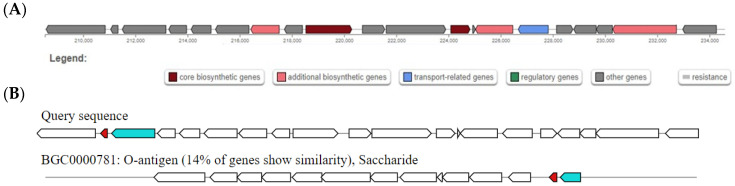
Thiopeptide BGC in the whole genome sequence of *Escherichia coli* C91 as predicted by antiSMASH. (**A**) Diagrammatical representation of thiopeptide BGC in the genome of *E. coli* C91. (**B**) Comparison of known BGCs with the thiopeptide BGC in the whole genome sequence of *E. coli* C91 as predicted by antiSMASH. The figure represents a set of BGCs with genes that encode for proteins with a predicted function. These genes are also predicted in the query BGC. The query sequence is the putative thiopeptide BGC in the WGS of *E. coli* C91. The percentage represents the percentage of genes in the BGCs that have a significant BLAST hit to genes within the current region. All figures were adapted from antiSMASH version 7.1.0.

In addition, the antiSMASH predicted one thiopeptide BGC in the genome of *E. coli* C91 to carry genes that have the potential to encode proteins involved in the production of a metabolic product related to O-antigen (Figure 6A). Only 14% of the genes were similar to the thiopeptide BGC in the WGS of *E. coli* C91 (Figure 6B).

## 3. Discussion

Dissemination of MDR bacteria is one of the most intricate public health crises. Many bacteria, including *Staphylococcus aureus*, *Klebsiella pneumoniae* and *Acinetobacter baumannii*, have become resistant to several classes of antibiotics, rendering antibiotic treatment ineffective [29]. Thus, there is an urgent requirement to discover new antibiotics [30]. In the present study, a clinical isolate of *E. coli* C91, previously overlooked as an antibiotic producer, was used for the detection of potential antibacterial compounds and siderophores. By integrating genome mining with ribosome engineering-type methods and gene deletion, this study presented evidence that *E. coli* C91 harbors cryptic biosynthetic potential but only activated after mutational perturbations and environmental stress. Several techniques can be used to enhance the antibiotic production capability of certain bacterial strain by awakening the silent BGCs. Some of these techniques include cultivation-based approaches such as changing environmental cues, co-cultivation, while molecular approaches include epigenetic mining, deletion of suppressors, ribosome engineering and mutagenesis [31].

The *rpoB* gene is responsible for encoding the β-subunit of RNA polymerase, and mutations in its rifampin resistance-determining region (RRDR) are a common way to induce drug resistance [31]. Rifampicin-resistant mutations alter transcription and indirectly reprogram global gene expression and metabolic pathways. Such *rpoB* mutations are often grouped under ‘ribosomal engineering’ strategies in the context of antibiotic discovery [32]. This is because these mutations specifically arise from selection with ribosome-targeting antibiotics and often change information-processing machineries in bacteria [33].

But, in addition to resistance, rpoB mutations can have wide-ranging transcriptomic effects, such as altered sigma factor usage and deregulation of metabolic pathways [32]. Spontaneous rifampicin-resistant *rpoB* mutants were generated in and provided a key result in this study. Unlike the wild type, the single nucleotide variant strain *E. coli* C91-R1 (S531L substitution) showed the ability to produce antibacterial metabolites against MRSA strain according to disk diffusion assays. This finding is consistent with a recent study that demonstrated the important role of global transcription perturbations in the expression of cryptic BGCs [33]. Moreover, this finding is also in agreement with earlier reports that confirmed the reprogramming of transcriptional networks in rifampicin resistance mutations in *rpoB* to awaken silent BGCs [34]. The mutation-specific nature of BGCs activation in the present study is reinforced by the increased activity in C91-R1, but not C91-R2 and C91-R3. This indicates that the S531L position may exhibit conformational effects on RNA polymerase [35]. Distinct transcriptional outcomes are caused due to different amino acid substitutions in *rpoB*, which exert specific structural and functional effects on bacterial RNA polymerase. Substitutions at S531 have been reported to alter the geometry of the β-subunit and affect (1) the stability of the RNA-DNA hybrid, (2) initiation dynamics and (3) open complex formation [36]. These changes can activate transcription of silent or “cryptic” BGCs through alternative sigma factor usage and modifying promoter selectivity [37]. However, mutations identified in C91-R2 and C91-R3, which are also RRDR mutations, induce different structural perturbations which oppose transcription of SM biosynthetic pathways. Like our report, others have documented activation of cryptic BGCs through *rpoB* substitutions, especially at S531, leading to enhanced secondary metabolite production [27]. Therefore, the C91-R1 antibacterial phenotype likely reflects a specific conformational and regulatory shift in RNA polymerase produced by the S531L mutation.

This phenotypic contrast is further reinforced with LC-MS/MS profiling of culture supernatants, where unique ion signatures for each mutant were detected. Only *m*/*z* 410.5 was observed in C91-R1 and a fragmentation pattern matching a polyene or NRB backbone was obtained. The molecule was not recognized by GNPS or METLIN database and is believed to be an unknown compound with antimicrobial activity. To fully characterize the structure and bioactivity of the molecule, purification, NMR characterization and cytotoxicity assays will be performed in the future. Moreover, C91-R2 and C9-R3 mutants resulted in discrete peaks, showing the BGC transcriptional levels for each mutant. This result confirms the ability of ribosome-engineering-type approaches to unlock silent biosynthetic pathways [36].

The impaired growth of the Δ*ycao* mutant under iron-deficient conditions was further proven using CAS assay, where siderophore production of the Δ*ycao* strain was significantly reduced (*p*-value). This implies that *ycao* has a regulatory function in the expression of siderophore biosynthesis or siderophore secretion [38]. Also observed in this phenotype was the transcriptional downregulation of *entB* (gene encoding for enterobactin biosynthesis), *mcmA* and *mchF* (genes involved in microcin maturation and export). Suppression of these genes in Δ*ycao* may hint at a potential role of *ycao*’s interbacterial competition and nutrient uptake regulation, although this point will need to be further investigated.

The simultaneous effect of *ycao* on siderophore and microcin-associated genes remains to be explored. Given that YcaO-family proteins usually function in peptide modification, Fur regulation, oxidative and envelope stress pathways can be the cause of this association [39]. Moreover, microcin operons are co-regulated with iron availability and global stress regulators rather than a single transcriptional factor [40]. Our current study therefore demonstrates simultaneous downregulation of *entB*, *mcmA* and *mchF* in the Δ*ycao* mutant, which can suggest disruption of integrated iron-dependent regulatory networks instead of direct transcriptional control by *ycao*. Thus, this study cannot confirm the link between siderophore biosynthesis and microcin pathways. However, this study observed transcriptional changes that can represent broader regulatory effects caused by loss of *ycao*, which influences iron homeostasis and stress [41]. Global regulatory effects have been previously reported for genes involved in iron uptake and SM biosynthetic pathways in *E. coli* [42].

We also observed impaired growth of Δ*ycao* in iron-chelated conditions, with no noticeable effect on growth under iron replete conditions. This indicates that *ycao* might function as a transcriptional repressor or co-regulator that can be derepressed in the presence of low iron, as previously confirmed with *Fur*-regulated genes [22]. Moreover, the significant growth reduction observed in Δ*ycao* under iron-limited conditions is consistent with previously established models in which scarce iron levels trigger global transcriptional adaptations that can reveal underlying regulatory defects [41]. These results confirm a possible relationship between siderophore and microcin biosynthesis pathways, further supporting the role of *ycao* as a regulatory node managing competitive antibacterial strategies and iron uptake [42]. However, our data are limited to gene expression and phenotype. Targeted biochemical assays and activity profiling will be required in future work to demonstrate the altered microcin production.

In this study, genome mining tools predicted that *E. coli* C91, an organism not known as an antibiotic producer, possesses cryptic BGCS, including NRP, thiopeptide and polyketide BGCs. Notably, the thiopeptide BGC exhibited low sequence similarity (14% identity) to the antiSMASH database BGCs. This suggests potential novel metabolite biosynthesis, which could be thiopeptide in structure. In agreement with prior research, our study also highlighted the antibiotic potential of well-characterized species and their potential to carry unexplored BGCs [11]. As shown in our study, mutational approaches and functional assays are capable of revealing metabolic pathways in under explored antibiotic-producing clinical isolates.

## 4. Materials and Methods

### 4.1. Bacterial Strain and Culture Conditions

The bacterial strain used in this study was *Escherichia coli* C91. This clinical strain was isolated from a post-surgical wound of a 53-year-old male patient [28]. This strain demonstrated antibiotic resistance to colistin (MIC > 32 mg/L) and intermediate resistance to imipenem and meropenem (MIC = 8 mg/L) [23]. The whole genome sequence (WGS) of *E. coli* C91 was accessed through Genbank. Genbank accession: SAMN10105215, ID: 10105215 (Sample name: *Escherichia coli* strain Kuwait C91).

### 4.2. Assays for Antibiotic Production by E. coli C91

*E. coli* C91 was screened for the production of SMs under various growing conditions using agar-well diffusion assays. For this purpose, two media (Luria broth and Nutrient broth), two temperatures (20 and 30 °C) and two shaking speeds (150 and 200 rpm) were chosen for the growth and cultivation of *E. coli* C91. Using each of the media mentioned, a small volume (10 mL) was prepared, and the bacterium was cultivated following the temperatures and shaking speeds mentioned above. The bacterial culture was then centrifugated at 1500 rpm for 20 min to separate the supernatant and bacterial pellet [43]. Membrane filters (0.22 µM pore size) were used to collect and filter the supernatants. These cell-free supernatants (CFS) were stored at −20 °C until further investigation.

#### 4.2.1. Agar-Well Diffusion Assays

Antibacterial activity of the CFSs was tested against a group of Gram-positive and Gram-negative indicator bacteria using agar-well diffusion assays [44] (Table 6). The indicator bacteria were cultivated, and the inocula were adjusted to a 0.5 McFarland. These inocula were spread onto Mueller–Hinton agar to create a uniform bacterial lawn. Wells of 8 mm in diameter were created using a 1 mL sterile disposable pipette tip. A volume of CFS (100 µL) was added to each well. For negative controls, wells were filled with sterile Luria broth or nutrient broth. For positive control, wells contained ciprofloxacin (10 µg/mL) for *K. pneumoniae* ADA100 and *A. baumannii* ADA155, whereas linezolid (10 µg/mL) was used for *S. aureus* Y27, *S. aureus* TCC 33592 and *S. agalactiae* ATCC 13813. Negative controls should produce no inhibition and positive controls should produce inhibition zones to confirm assay performance. The plates were incubated for 24 h at 37 °C. The diameter of the zones of inhibition was measured following the incubation period, and no zone of inhibition was reported as having no inhibitory activity (8 mm) [45].

#### 4.2.2. Spot-on-Lawn Assay

Wild-type and mutant strains of *E. coli* C91 were screened to verify the presence of antimicrobials using the spot-on-lawn assays [46]. Cultures of *E. coli* C91 were grown in 5 mL nutrient broth at 37 °C for 24 h. Lawns of indicator bacteria were prepared on nutrient agar, as mentioned in part 2.2.1 of this study. The plates were then spotted with 2 µL of culture broth of *E. coli* C91 and its mutants derived from this study. These plates were incubated for 24 h at 37 °C. Inhibition was determined if a zone of clearing (>3 mm) was observed around the producer colony. This assay was repeated in triplicate.

### 4.3. Determination of MICs

Minimum inhibitory concentration (MIC) of rifampicin (Sigma-Aldrich, Dorset, UK) was determined in this research by the broth microdilution assays according to CLSI guidelines [47]. The lowest concentration of the tested compound that prevented growth was recorded as the MIC. *Escherichia coli* ATCC 25922 was used as a reference strain.

### 4.4. Selection of Rifampicin-Resistant E. coli C91 Mutants

After determining the MIC of rifampicin to *E. coli* C91 was 1 µL, 10 ug/mL rifampicin (10X the MIC) was used for selecting resistance mutants in *E. coli* C91. These mutants were needed for this study to enhance the production of antibiotics in *E. coli* C91 [29]. *E. coli* C91 suspension (10^6^ CFU/mL) was spread on nutrient agar plates supplemented with 10 µg/mL of rifampicin. The plates were incubated at 37 °C and checked daily for the presence of colonies up to 5 days. Three (3) candidate rifampicin-resistant *E. coli* C91 colonies were picked and named *E. coli* C91-R1, C91-R2 and C91-R3. Bacterial cultures were grown and stored for further examination.

### 4.5. DNA Extraction, Amplification and Sequencing

The full-length rpoB gene sequence was amplified from genomic DNA of *E. coli* C91 using three overlapping primer sets: rpoB-F1 (5′-GCGGCTCAGCGGTTTAGTTG-3′) and rpoB-R1 (5′-ACAGCGGGTTGTTCTGGTCC-3′), rpoB-F2 (5′-GACGACATCGACCACTTC-3′) and rpoB-R2 (5′-GATCACCTTGCCGGATTC-3′). Polymerase chain reactions (PCR) were performed with high-fidelity DNA polymerase according to the following scheme of thermal cycling conditions: denaturation at 94 °C for 10 min; 35 cycles of 94 °C for 1 min, annealing at 57 °C for 40 s and extension at 72 °C for 50 s and a final extension step was carried out at 72 °C for 10 min [48]. The PCR products were purified, dried and then sequenced with the ABI 3500xl Genetic Analyzer (Applied Biosystems, Foster City, CA, USA). The reads were mapped to the *Escherichia coli* K-12 MG1655 reference genome (GenBank accession no. NC_000913.3) by using BioEdit v7.0.5.3.

### 4.6. Analysis of Antibiotic Production by Mutant Strains of E. coli C91

All three mutants of *E. coli* C91 were screened for the production of antibiotics and were compared to that of the wild type. These mutants were grown in 10 mL Luria broth at 30 °C shaking at 150 rpm. The cultures were centrifuged for 20 min at 1500 rpm. The CFSs were then collected using 0.22 µM pore size membrane filters. Antibacterial activity of these supernatants was determined using agar-well diffusion assays, as performed in Section 4.2.1.

### 4.7. Generation of ycao Deletion Mutant in E. coli C91

An in-frame deletion of the *ycao* gene in *E. coli* C91 was generated by the λ Red recombinase-mediated allelic exchange method using site-specific recombinases of phage λ, as described by Datsenko and Wanner, with slight modifications [49]. The PCR product containing a kanamycin resistance cassette with FRT sites was generated from plasmid pKD4 through amplification using primers that included 50 bp sequences matching the upstream and downstream regions of the *ycao* gene (Supplementary Data Table S1). The PCR products were electroporated into *E. coli* C91 (bearing the temperature-sensitive plasmid pKD46 encoding λ Red recombinase from an arabinose-inducible promoter). Transformant selection was performed on the Luria broth agar plate supplemented with kanamycin at 50 μg/mL, and the corresponding colony PCR was conducted with the flanking primers of the *ycao* locus for the positive transformants. The FLP recombinase from plasmid pCP20 removed the resistance cassette. The final mutant (Δ*ycao*) underwent PCR and Sanger sequencing to verify both precise deletion and marker removal.

### 4.8. Chrome Azurol (CAS) Assay

Chrome Azurol S (CAS) assay detects iron-chelating activity through a visible color transformation of a blue indicator dye. It was performed in this research to evaluate siderophore production by *E. coli* C91 and its Δ*ycao* mutant.

The CAS agar diffusion method was performed as described by Schwyn and Neilands, with minor alterations [50]. The stability of CAS reagent was checked prior to use to confirm if the CAS indicator was responsive throughout the assay. To confirm if the CAS-Fe^3+^ complex retained its typical blue color without random orange discoloration, uninoculated plates were incubated at 30 °C for 24–48 h. As a positive control, *Pseudomonas aeruginosa* PAO1, a known siderophore producer, was included and was producing a distinct orange halo. Sterile medium was used as the negative control and remained blue.

*E. coli* C91 wild type and Δ*ycao* mutant strains were grown overnight in Luria broth and were adjusted to 1.0 OD_600nm_. From this suspension, 5 mL were spotted onto CAS plates, followed by incubation at 30 °C for 48 h. To quantify siderophore activity, diameters of orange halos surrounding the colonies were measured in millimeters (mm). Each strain was tested in triplicates and values were reported as mean halo diameter ± s.d. The relative siderophore activity was calculated as a percentage of wild-type levels.

### 4.9. Growth Assays Under Iron-Limiting Conditions

Growth of *E. coli* C91 wild type and Δ*ycao* mutant strains was studied when iron levels were restricted. *E. coli* C91 wild type and Δ*ycao* mutant strains were grown in M9 minimal medium containing 0.2% glucose and 2 mM MgSO_4_ and 0.1 mM CaCl_2_. To establish iron-limiting conditions in the medium, 100 µM 2,2′-dipyridyl (Sigma-Aldrich) was added as a ferrous iron chelator to create iron-limited conditions [51]. Control cultures comprising the same medium excluding the iron chelator. Two consecutive wash cycles were established for the initial cultures with M9 medium, followed by dilution to 0.01 OD_600nm_ in either iron-limited medium or control medium. The bacterial cultures were incubated at 30 °C in a 96-well plate while FLUOstar Omega™ spectrophotometer (BMG LABTECH, Ortenberg, Germany) recorded the optical density (OD_600nm_) readings every 30 min for 15 h. The generated growth curves received area under the curve (AUC) analysis for quantitative assessment. This assay was repeated in triplicates.

### 4.10. LC-MS Analysis of Culture Supernatant

Solvent extracts of CFSs from *E. coli* C91 wild type, and rifampin-resistant mutants (C91-R1, C91-R2 and C91-R3) were cleaned using a Sep-Pak tC18 Plus long-cartridge solid-phase extraction (SPE) column (Fisher Scientific, Cleveland, OH, USA). The bacterial strains were incubated in 50 mL Luria broth at 30 °C and shaking at 150 rpm overnight. The pellets from the centrifugation (10,000× *g*, 10 min at 4 °C) were discarded and the supernatants were collected and filtered through 0.45 µM filter units. SMs were collected from the surfaces of the extracts with ethyl acetate at a ratio of 1:1 for 24 h on a rolling shaker [52]. The organic layer was combined and evaporated to dryness at 45 °C using a rotary evaporator, reconstituted in 500 µL HPLC-grade methanol. Each sample was frozen at −80 °C until analysis by LC-MS/MS.

Profiling of the extracted metabolites was performed using a liquid chromatography system (Agilent 1290 Infinity II) with a coupled QTOF mass spectrometer (Agilent 6545 QTOF) equipped with an ESI source (version B.08.00, Agilent Technologies, Santa Clara, CA, USA).

LC-MS was performed under the following conditions: 5 µL of sample was injected into C18 reversed-phase analytical column (2.1 × 100 mm, 1.7 µm), which was used for LC separation. The mobile phase was composed of (A) water with 0.1% formic acid and (B) acetonitrile + 0.1% formic acid. Over 15 min, a gradient elution was applied from 5 to 95% B at a flow rate of 0.3 mL/min. The capillary voltage was set at 2.5 kV, cone voltage at 40 V, source temperature at 120 °C, desolvation temperature at 450 °C and desolvation gas flow at 800 L/h. Data was acquired in positive-ion mode across a mass range of *m*/*z* 100–1500. MS/MS spectra were collected using a collision energy ramp at 15–45 eV.

The mass spectral data were processed by Agilent MassHunter Qualitative Analysis software (version B.07.00, Agilent Technologies, Santa Clara, CA, USA). Comparing Base peak chromatograms (BPCs) and extracted ion chromatogram (EIC) analyses showed some peaks that were detected in mutant strains, but not in the wild type [53]. The GNPS and METLIN databases were searched based on the accurate mass searches to tentatively identify compounds. Virtual fragmentation prediction tools were used to simulate MS/MS spectra of precursor ions chosen for structural analysis. In consistency with recommendations for exploratory metabolomics workflow, LC-MS data was interpreted qualitatively, and metabolite abundance was not subject to statistical comparison [24].

### 4.11. Transcriptional Analysis by Real-Time qPCR

Transcriptional analysis of *Ycao* gene in wild type and the mutant (Δ*ycao*) *E. coli* was conducted by RT-PCR. Total RNAs were prepared using Total RNA Ultrapure Kit (Creative Biogene, Shirley, NY, USA) according to the manufacturer’s protocol. Real-time quantitative (qPCR) was performed as described previously [54]. The total RNAs (1 µg) was treated with RNA-free DNase I (Invitrogen, Carlsbad, CA, USA) and was subsequently used as a template for reverse transcription (20 µL) using a high-capacity RNA-to-cDNA Kit (Applied Biosystems, Foster City, CA, USA). qPCR was performed using the ABI7500 real-time PCR system (Applied Biosystems) and THUNDERBIRD qPCR Mix (Toyobo Co., Ltd., Osaka, Japan) in triplicate. All PCR procedures were performed under the following conditions: 95 °C for 5 min, followed by 40 cycles of 15 s at 95 °C for denaturation, followed by annealing and extension for 40 s at 60 °C.

Each qPCR reaction was performed in a final volume of 20 µL containing 10 µL of 2× SYBR Green master mix, 0.4 µM of each primer and 2 µL of diluted cDNA template. Gene-specific primers used in this study are listed in Supplementary Table S1. Primer pairs were designed using Primer-BLAST to ensure target specificity and lack of off-target amplification. Primer efficiencies were verified and fell within the acceptable range of 90–110%. A single amplification product for each reaction was confirmed by melt-curve analysis. All assays were performed in replicates.

### 4.12. In Silico Prediction of Biosynthetic Gene Clusters

Using multiple genome mining tools, conserved biosynthetic genes were identified to predict secondary metabolite biosynthesis pathways coding for compounds with probable antibiotic action. The WGS of *E. coli* C91 was submitted to three genome mining platforms: antiSMASH, PRISM and BActeriocin GEnome mining tool (BAGEL4). The antiSMASH was accessed through the website: https://antismash.secondarymetabolites.org/ (accessed on 16 July 2023). To detect well-defined clusters, the detection strictness was set to “strict”. All of the extra parameters, including KnownClusterBlast, ActiveSiteFinder, ClusterBlast, Cluster Pfam analysis, SubClusterBlast and Pfam-based GO term annotation, were enabled [45]. BAGEL4 was accessed at: http://bagel.molgenrug.nl/ (accessed 16 July 2023). The WGS was also submitted to BAGEL4 by loading the WGS file in FASTA format. The webpage https://github.com/magarveylab/prism-releases (accessed on 16 July 2023)was used to access PRISM [46]. The WGS of *E. coli* C-91, potentially coding for proteins involved in antibacterial biosynthesis, was submitted to PRISM by loading sample input in FASTA format [12]. No other changes were made as all the ‘Advanced settings’ options were pre-selected.

### 4.13. Statistical Analysis

All experiments were performed in triplicates, and the results were expressed as ±standard error. Statistical Packages for Social Sciences (SPSS) software version 24 was used to statistically analyze the data (SPSS Inc., Chicago, IL, USA). Growth curve: growth of *E. coli* C91 (wild type) and Δ*ycao* (mutant) were statistically compared using unpaired two-tailed Student’s *t*-tests. Differences between means of area under the growth curve (AUC) were considered statistically significant if *p*-value ≤ 0.05.

## 5. Conclusions

Our results reveal hidden biosynthetic potential in *E. coli* C91 through the conjugation of antimicrobial screening, LC-MS/MS and gene knock out with selection of rifampicin-resistant mutants. The putative novel compound (*m*/*z* 410.5) found exclusively in C91-R1 is an interesting result that requires further elucidation as to its structural and cytotoxicity activity. Furthermore, this work uncovered the bifunctional role of the *ycao* gene in siderophore biosynthesis and microcin-associated pathways, highlighting its critical functions in interbacterial competition and iron scavenging. Altogether, with the current epidemic of antimicrobial resistance, *E. coli* C91 may provide hope for increasing the arsenal of antibiotics to combat antibiotic resistance.

## Figures and Tables

**Table 1:** Antibacterial activity of *E. coli* C-91 and mutants.

Strain	Zone of Inhibition ^a^ (mm)		Rifampicin Resistance (µg/mL)	Mutation Detected (*rpoB*)	Reference or Source
	*S. aureus* Y27	*S. aureus* ATCC 33592			
*E. coli* C91	0 ± 0	0 ± 0	<5	None	[28]
*E. coli* C91-R1	19 ± 0.72	20 ± 0.59	>200	S531L	This study
*E. coli* C91-R2	0 ± 0	0 ± 0	>200	H526Y	This study
*E. coli* C91-R3	0 ± 0	0 ± 0	>200	D516V	This study

^a^ The strains were grown in Luria broth at 30 °C for 24 h while shaking at 150 rpm. Antibacterial activity of the supernatants against *S. aureus* Y27 and *S. aureus* ATCC 33592 was determined by agar-well diffusion assays. Experiments were conducted in triplicates, and the data is expressed as mean ± standard deviations.

**Table 2:** LC–MS profiling.

Sample	New Peaks Detected (*m*/*z*)	Putative Compound Matched
*E. coli* C91 (WT)	None	-
*E. coli* C91-R1	410.5	Non-ribosomal peptide-like compound
*E. coli* C91-R2	347.2, 364.3	Possible polyketide or peptide
*E. coli* C91-R3	297.1	Novel metabolite (no database match)

**Table 3:** Evaluation of siderophore production by wild-type *E. coli* C91 and the Δ*ycao* mutant.

Strain	Siderophore Production (mm)	Relative Siderophore Units (RSU) *
*E. coli* C91 (wild type)	14.2 ± 0.4	100%
Δ*ycao* (mutant)	5.6 ± 0.3	39%

Siderophore production was presented as mean ± SD. * *p*-Value of the mutant versus the wild type = 0.0002.

**Table 4:** Growth patterns of *E. coli* C91 and Δ*ycao* mutant strain under different iron conditions.

Growth Condition	Strain	Final OD_600nm_	AUC
Iron replete (control)	*E. coli* C91 (WT)	1.12 ± 0.04	9.11 ± 0.12
	Δ*ycao* mutant	1.09 ± 0.03	8.88 ± 0.23 *
Iron-limited	*E. coli* C91 (WT)	1.0 ± 0.04	8.89 ± 0.15
	Δ*ycao* mutant	0.59 ± 0.05	5.54 ± 0.35 **

OD: optical density, AUC: area under the curve. * Under iron-repleted conditions, the difference between wild type and Δ*ycao* mutant was not statistically significant (*p* = 0.48). ** Under iron-limited conditions, the reduction in growth of Δ*ycao* mutant was significant (*p* = 0.0001, unpaired two-tailed *t*-test). Final OD_600nm_ and AUC values are presented as mean ± SD (*n* = 3).

**Table 5:** Putative biosynthetic gene clusters in the WGS of *E. coli* C-91.

Tool	Cluster ^a^	Type	Most Similar Known Cluster ^b^	Similarity (%)
antiSMASH	1	NRP	Enterobactin BGC	100
			Frederiksenibactin BGC	69
			Trichryobactin BGC	69
			Photobactin BGC	41
antiSMASH	2	Thiopeptide	O-antigen BGC	14
PRISM	1	Polyketide	-	-

^a^ Cluster represents the order of BGC predicted by the tool. ^b^ Most similar known clusters are predicted by antiSMASH, starting from the one with highest % similarity to lowest similarity %. NRP; non-ribosomal peptide, -; not predicted by PRISM.

**Table 6:** Indicator bacteria used in this study and their phenotypes.

Indicator Organism	Phenotype of Resistance *	Institute/Company
*Klebsiella pneumoniae* ADA 100	AMP, COL, CAZ, TET	Medical Laboratory Sciences, Faculty of Allied Health Sciences, Kuwait University
*Acinetobacter baumannii* ADA 155	AMP, CTX, CAZ, CH, TET	
*Staphylococcus aureus* Y27	MET, VA	
*Streptococcus agalactiae* ATCC 13813	GM, MET	American Type Culture Collection (ATCC)
*Staphylococcus aureus* ATCC 33592	VA, CIP, GM, MET	

* Abbreviations correspond to resistance to VA, vancomycin; CIP, ciprofloxacin; GM, gentamicin; MET, methicillin; COL, colistin; TET, tetracycline; CAZ, ceftazidime; AMP, ampicillin; CTX, cefotaxime; CH, cephalothin.

## Data Availability

The original contributions presented in this study are included in the article/Supplementary Materials. Further inquiries can be directed to the corresponding author.

## Supplementary Materials

The following supporting information can be downloaded at: https://www.mdpi.com/article/10.3390/antibiotics15010043/s1, Table S1: Primer sequences used for generation and confirmation of Δ*ycao* mutant and ampli fication of iron-associated and microcin-associated genes; Table S2: Nucleotide sequences of wild type and Δ*ycao* loci in *E. coli* C91; Table S3: Raw qRT-PCR results corresponding to the bar graph.

## Abbreviations

The following abbreviations are used in this manuscript:

antiSMASH	Antibiotics and Secondary Metabolites Analysis Shell
ATCC	American Type Culture Collection
AUC	Area under the curve
BAGEL4	BActeriocin GEnome mining tool
BGC	Biosynthetic gene cluster
BPC	Base peak chromatograms
CAS	Chrome Azurol S
CDC	Centers for Disease Control and Prevention
CFS	Cell-free supernatant
CNF1	Cytotoxicity necrotizing factor 1
HPLC	High-performance Liquid Chromatography
EIC	Extracted ion chromatogram
Fur	Ferric uptake regulator
MDR	Multidrug-resistant
MIC	Minimum inhibitory concentration
MRSA	Methicillin-resistant *Staphylococcus aureus*
NP	Natural product
PRISM	PRediction Informatics for Secondary Metabolomes
rpm	Rotations per minute
qRT-PCR	Quantitative real-time PCR
SM	Secondary metabolite
SPE	Solid phase extraction
SPSS	Statistical Packages for Social Sciences
UPEC	Uropathogenic *E. coli*
VRE	Vancomycin-resistant enterococci
WGS	Whole genome sequence
WHO	World Health Organization
