# Supplementary material for: Functional Characterization of ycao in Escherichia coli C91 Reveals Its Role in Siderophore Production, Iron-Limited Growth, and Antimicrobial Activity
Source: Antibiotics (Basel). 2026 Jan 1;15(1):43. doi: 10.3390/antibiotics15010043 (PMC12837419; doi:10.3390/antibiotics15010043)
Supplement: Supplementary file 1 [file antibiotics-15-00043-s001.zip › antibiotics-4029277-supplementary.pdf]

Supplementary data

Table S1. Primer sequences used for generation and confirmation of Δ*ycao* mutant and ampli fication of iron-associated and microcin-associated genes.

Gene	Primer name	Sequence (5′→3′)	Amplicon size (bp)*
entB	*entB*_qF	**AGCTGACCATCGTTACCGTT**	~120
entB	*entB*_qR	**TTCGATGATGCCGATACGAC**	
mcmA	*mcmA*_qF	**CGTCTGATGATGCTGCTGTT**	~135
mcmA	*mcmA*_qR	**ACGTTGATCGGCTTGATGAC**	
mchF	*mchF*_qF	**GCTGATCGTTGCTGATGCTG**	~140
mchF	*mchF*_qR	**TTGACGATCAGCGTAGTCGC**	
ycao	*ycao*_qF	**ATGCTGACCGTTATCGACGA**	~130
ycao	*ycao*_qR	**CGATGATCGGCAATACCTGC**	

Table S2. Nucleotide sequences of wild type and Δ*ycao* loci in *E. coli* C91.

Strain	Region	Sequence (5′→3′)	Notes
WT	Full *ycao* locus	ATGAACTACGCGGATC…[~1115 bp]	Includes full 615 bp *ycao* CDS
Δ*ycao*	Deleted locus	ATGAACTACGCGGATC…[~500 bp]	Fusion of upstream/downstream ends, in-frame deletion

Table S3. Raw qRT-PCR results corresponding to the bar graph

Gene	WT (Mean)	WT ± SEM	Δ*ycao* (Mean)	Δ*ycao* ± SEM	P-value
entB	1	1.00 ± 0.05	0.45	0.45 ± 0.04	0.01
mcmA	1	1.00 ± 0.06	0.2	0.20 ± 0.02	0.0005
mchF	1	1.00 ± 0.04	0.7	0.70 ± 0.03	0.08
